# Editorial Board of the West of England Medical Journal AGM

**Published:** 1992-08

**Authors:** P. Goddard, J. R. Farndon

**Affiliations:** Chairman of Editorial Board


### Editorial Board of the West of England Medical Journal AGM

The Editorial Board of the West of England Medical Journal held its Annual General Meeting on Wednesday 15 July 1992 at the Forte Posthouse Hotel in Deane Gate Avenue, Taunton. Twelve members of the Editorial Board were at the meeting.

Readers will know from previous editorial material of the parlous financial plight of the Journal and that only one more issue of the Journal will be possible with our current finances. Many attempts have been made to obtain financial stability and viability for the future. This has not been forthcoming from any of the clubs or groups associated with the Journal, other than the Surgical Club of South West England who were willing to raise their subscriptions by £10 per head. Other clubs and societies made generous offers of financial support as one off payments but these in themselves came nowhere near to meeting the financial requirements of a journal which tries to produce four issues each year.

The Editorial Comittee has made long and hard attempts to obtain money from advertising. It has approached three publishing houses which gave some hope of being able to publish the Journal on advertising revenue but these offers have not come to fruition.

At the Editorial Committee Meeting, therefore, it was decided that the Journal would close following the publication of the last two issues in 1992.

The Committee resolved to ask all recipients of the Journal whether they would be willing to subscribe as individual recipients. The suggested subscription would be £20 per annum. If 1,000 recipients or more were willing to pay this subscription the Journal would be viable and other issues would be presented in 1993. If less than 1,000 were willing to subscribe the Journal would close. You will find a tear off slip at the bottom of this page which you should use to write to the Chairman of the Editorial Board. The Editorial Committee would urge you to respond and hopefully respond positively. This is the last chance that you will have to save one of the oldest running medical journals in the United Kingdom.

The Editorial Committee thank you for your support and help in the past. The Committee hopes that you will respond favourably and with offers of support.

P. Goddard, Editor
J. R. Farndon, Chairman of Editorial Board

---

**Please return this slip to:—**

**THE CHAIRMAN,**

**EDITORIAL BOARD OF THE**

**WEST OF ENGLAND MEDICAL JOURNAL,**

